# The art of fin regeneration in zebrafish

**DOI:** 10.1002/reg2.33

**Published:** 2015-05-19

**Authors:** Catherine Pfefferli, Anna Jaźwińska

**Affiliations:** ^1^ Department of BiologyUniversity of FribourgCh. du Musée 101700FribourgSwitzerland

**Keywords:** blastema, caudal fin, epigenetics, regeneration, zebrafish

## Abstract

The zebrafish fin provides a valuable model to study the epimorphic type of regeneration, whereby the amputated part of the appendage is nearly perfectly replaced. To accomplish fin regeneration, two reciprocally interacting domains need to be established at the injury site, namely a wound epithelium and a blastema. The wound epithelium provides a supporting niche for the blastema, which contains mesenchyme‐derived progenitor cells for the regenerate. The fate of blastemal daughter cells depends on their relative position with respect to the fin margin. The apical compartment of the outgrowth maintains its undifferentiated character, whereas the proximal descendants of the blastema progressively switch from the proliferation program to the morphogenesis program. A delicate balance between self‐renewal and differentiation has to be continuously adjusted during the course of regeneration. This review summarizes the current knowledge about the cellular and molecular mechanisms of blastema formation, and discusses several studies related to the regulation of growth and morphogenesis during fin regeneration. A wide range of canonical signaling pathways has been implicated during the establishment and maintenance of the blastema. Epigenetic mechanisms play a crucial role in the regulation of cellular plasticity during the transition between differentiation states. Ion fluxes, gap‐junctional communication and protein phosphatase activity have been shown to coordinate proliferation and tissue patterning in the caudal fin. The identification of the downstream targets of the fin regeneration signals and the discovery of mechanisms integrating the variety of input pathways represent exciting future aims in this fascinating field of research.

“*J'ai remarqué que les nageoires se réparoient d'ordinaire plus ou moins promptement*

*suivant qu'elles étoient plus ou moins utiles à l'animal*.” (Broussonet 1786)

“*I remarked that the fins were renewed generally sooner or later, according as they were more or less useful to the animal*.” (Broussonet 1789)

## The Discovery of Fin Regeneration

The teleost fish, together with urodele amphibians, represent unique vertebrates with a spectacular capability to regenerate various organs after traumatic injury. For both animal groups, regenerative biology research was initiated by studying the external appendages. This is not surprising, because amputation of fins or limbs and the documentation of their regrowth can be achieved by simple manipulations and macroscopic observations, whereas similar procedures are less evident for internal organs. The first report about fin regeneration was written by the French naturalist Broussonet in 1786 in his native language, which was later translated into English (Broussonet 1786, 1789). Remarkably, this historical reference has been rather unnoticed in the current literature. Based on experiments with goldfish, Broussonet discovered fin regeneration, and importantly, he identified that the caudal fin displays experimental advantages over the other fin types because it regenerates more quickly than the ventral, pectoral, and dorsal appendages. Indeed, this finding still holds today, and current research continues to use the tail of fish to study the cellular and molecular mechanisms underlying organ restoration in vertebrates. Thus, the caudal fin as a model system has a remarkably long history of at least 230 years, and the author of the pioneering study deserves to be considered as the father of fish regenerative biology.

One of the important, and still unsolved, questions from Broussonet's work concerns the correlation between the regrowth rate, the fin type and amputation position. Which mechanisms regulate the rate of regeneration of anatomically similar structures? Broussonet noticed that the efficiency of regeneration was correlated with the functional importance of the lost fin surface for the swimming performance. This phenomenon was then reinvestigated by Morgan, who at the beginning of the 20th century described the shape of the outgrowths after asymmetrical cutting of fins in various fish species (Morgan 1900, 1902, 1906). Morgan's experiments showed a gradient of regeneration rate along the proximo‐distal axis with the highest values at the base of the fin. He assumed that the mechanisms underlying this gradient could not be of typical physiological nature, because the histological and anatomical features within the entire fin are nearly identical. In agreement with Broussonet's hypothesis, Morgan proposed that certain “formative factors” increase the growth rate at the positions “where most material is needed to complete the typical form of the tail” (Morgan 1902). Moreover, he hypothesized that “the new material assumes the typical form before it has reached its full size” (Morgan 1900). This long‐standing conceptual interest in the regulation of regenerative growth and patterning has been readdressed in the last few years in the zebrafish model organism using modern molecular biology tools and genetics. In this review, we describe the fin as a model system and the recent findings related to the classical questions about growth and morphogenesis during regeneration.

## The Fin, a Non‐Muscularized Dermal Appendage

The zebrafish is the fish species most commonly used as a model organism in current biomedical research (Kari et al. 2007; Brittijn et al. 2009; Gemberling et al. 2013; Tavares & Santos Lopes 2013). As in goldfish, the zebrafish caudal fin has several ideal properties for experimental procedures and regeneration studies. First, it is the largest external appendage located at the posterior end of the body, which makes it the most accessible for surgery and imaging. Second, in contrast to the remaining fins, it displays a bi‐lobed morphology that is optimal for analysis of the differential growth rate along the medial−lateral axis. Third, the fin has some unique features compared to the amphibian limb. It exhibits a simpler anatomy, lacking certain tissues such as muscles and cartilage. Fourth, the completion of tail regeneration is rapidly and faithfully achieved within 2−4 weeks depending on the water temperature. Finally, rays can regenerate independently of each other, providing autonomous regenerative units and multiple experimental replicates within the same appendage (Nabrit 1929). These powerful features render the caudal fin an ideal model system to tackle fundamental issues concerning vertebrate organ regeneration.

**Figure 1:**
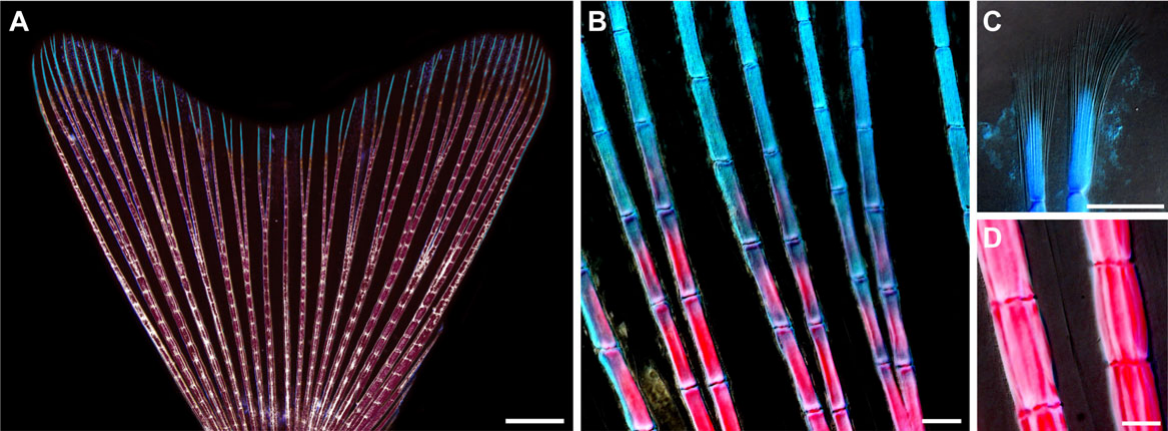
The skeleton of the zebrafish caudal fin. (A)−(D) Whole mount view of an adult caudal fin stained with Alcian Blue and Alizarin Red to visualize the skeleton. (A) The bi‐lobed morphology of the caudal fin fold is stabilized by 16−18 segmented and occasionally bifurcated bony rays (stained structures), named lepidotrichia, that are interconnected by soft interray tissue (unstained regions between the bones). The segmental borders contain ligaments with a regular spacing along the proximo‐distal axis (a whitish ladder‐like pattern of each ray). The bones are predominantly composed of calcified matrix (magenta), with the exception of the distal parts which remain non‐mineralized (cyan). (B) A higher magnification of the distal region shows a gradual decrease of the calcification level towards the fin margin. The length of segments is nearly identical in proximal (magenta) and distal (cyan) parts of the rays. (C) The tips of the rays are supported by a brush‐like bundle of fine spicules, called actinotrichia, which surround the apical‐most segment of the lepidotrichia and expand further distally beyond the end of the bone. (D) The proximal segments of the rays are at least three times broader than the distal calcified segments (compared with B), but their length remains nearly constant. Scale bars: (A) 1000 μm; (B)−(D) 100 μm.

The zebrafish caudal fin originates predominantly from the ventral side of the larval fin. During adulthood, it remains connected to the vertebral column by bones of ventral origin, with the exception of the dorsal‐most rays (Géraudie et al. 1995). Anatomically, this appendage can be defined as a non‐muscularized dermal fold that is stabilized by 16−18 main segmented and occasionally bifurcated rays spanned by soft interray tissue (Fig. 1A, B). The segment length is demarcated by the intersegmental joints that are spaced approximately 240−320 μm ranging from the distal to proximal terminus of the ray, the formation of which can be mathematically modeled (Rolland‐Lagan et al. 2012). The bi‐lobed shape of the adult fin arises as the result of a higher number of segments in the lateral rays of the lobes compared to the medial rays of the cleft, displaying a difference of approximately four segments between the longest and the shortest ray (Goldsmith et al. 2006). As fish can grow during their entire lifespan, fins maintain a capacity of extending their size throughout adulthood. The growth of the fin is achieved by the sequential addition of new ray segments at the tip which, once formed, can become increasingly thicker but cannot elongate (Goldsmith et al. 2006). Thus, in contrast to a tetrapod limb with a constant sequence of bones, which is set up during embryogenesis, the number of ray segments increases in proportion to the growth of the animal. Each newly grown ray segment arises as a distal unit, but it acquires a proximal value as the elongation of the tail continues. The same situation takes place for the ray bifurcations, which originate at the distal tip of the growing fin but become proximal after generation of new segments during ontogenetic growth (Goldsmith et al. 2006). Consequently, the proximo‐distal positional values are not intrinsic to a particular ray segment or bifurcation point, but this axis varies according to the actual dimension of the fin.

The robustness of the fin fold depends predominantly on the collagenous bone matrix, called lepidotrichia, which is deposited by osteoblasts (also named scleroblasts) underneath the epidermis. The major proximal portion of the ray is supported by calcified bone matrix, while the three to four distal‐most segments are thin and remain non‐mineralized (Fig. 1C, D). The gradient of ray mineralization indicates the smooth transition between the proximal and distal portion of the appendage (Fig. 1B). The distal‐most segment of each ray lacks bone matrix at the tip. However, it is supported by a brush‐like bundle of fine spicules, named actinotrichia (Fig. 1C), which are synthesized by non‐osteoblasts (Zhang et al. 2010; Durán et al. 2011). It is reasonable to assume that mineralized matrix at the base and flexible structures at the tip of the appendage provide optimal architecture for the hydrodynamic function of the fin.

**Figure 2:**
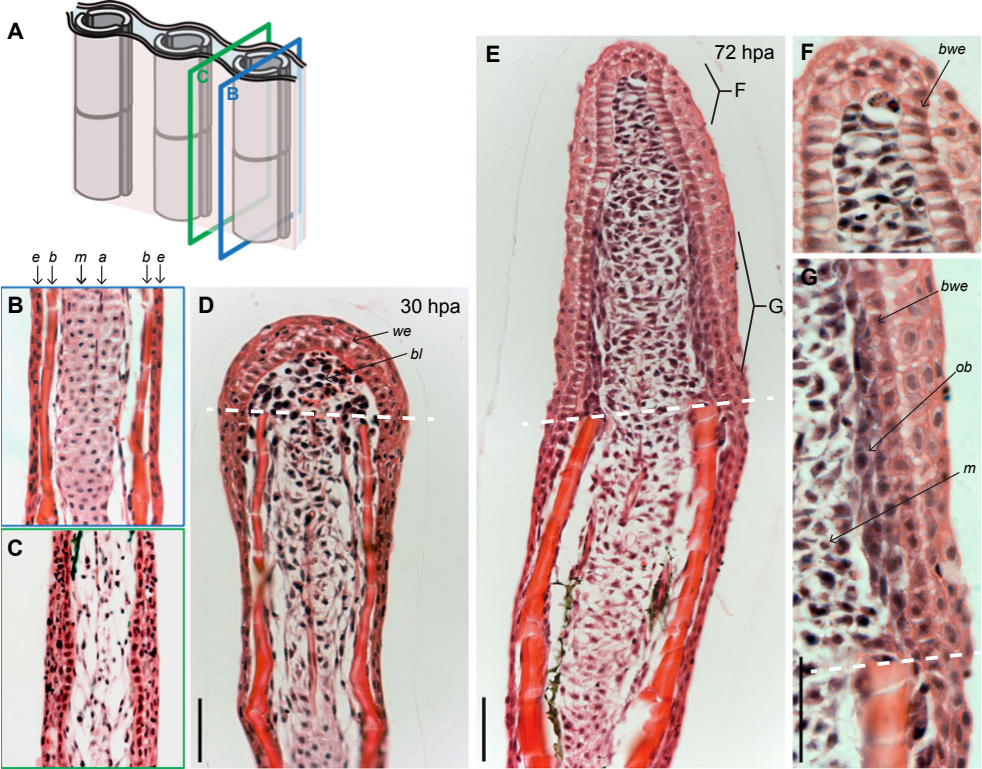
The histological organization of an uninjured and regenerating caudal fin. (A) Schematic representation of the fin structure with the planes of sectioning along the interray (green frame) and rays (blue frame). (B)−(I) Longitudinal fin sections stained with hematoxylin and eosin. (B) Each lepidotrichium consists of a pair of concave bones (*b*) that appear as parallel rods underneath the multilayered epidermis (*e*). Bones are tightly covered by flattened osteoblasts that deposit the bone matrix. The mesenchymal tissue (*m*) between the bones is composed of connective tissue containing densely interconnected fibroblasts, nerves, and arteries (*a*). (C) The interray is devoid of skeletal elements and contains loose connective tissue. (D) At 30 hpa, the blastema (*bl*) appears as a cluster of undifferentiated mesenchymal cells covered by a wound epidermis (*we*) above the amputation plane (white dashed line). Blastema formation results from the dedifferentiation of cells located in the stump that progressively lose their specialized morphology, initiate proliferation, and migrate distally toward the amputation plane. (E) At 72 hpa, the blastemal outgrowth exhibits a spatial organization of the newly formed tissue. (F) Higher magnification of the distal part of the outgrowth (apical signaling zone with slowly cycling cells). Mesenchymal cells become elongated perpendicularly to the growth (proximo‐distal) axis. The basal layer of the wound epithelium (*bwe*) contains columnar cells. (G) Higher magnification of the proximal part of the outgrowth (proliferation and redifferentiation zone). Dedifferentiated osteoblasts (*ob*) are tightly interconnected and remain aligned underneath the wound epidermis. The basal layer of the wound epithelium (*bwe*) contains cuboidal cells. The mesenchymal cells are round and loosely distributed. Scale bars: 50 μm.

The ray contains two concave bones at each side of the fin fold, called hemirays. The bilateral organization of the ray can be assessed in longitudinal fin sections (Fig. 2A). In this perspective the pair of concave bones appears as parallel rods below the multilayered epidermis (Fig. 2B). The lepidotrichia are tightly covered by flattened osteoblasts that deposit matrix to adjust the diameter of the bone during growth. The space between the hemirays is filled with connective tissue, which, in contrast to typical mammalian dermis, contains densely interconnected fibroblasts (Fig. 2B). The rays are innervated and vascularized by central arteries (Huang et al. 2003). The interrays, which separate adjacent rays, lack skeletal elements and contain veins embedded in a mesenchymal tissue with larger spacing between cells (Fig. 2C). Taken together, fins are composed of multiple tissues, including connective tissue, lepidotrichia, actinotrichia, blood vessels, nerves, and epidermis, all of which must regenerate coordinately to restore the shape and function of the organ. Direct interactions between adjacent tissues have to be established to synchronize the regrowth and patterning.

## Fundamental Properties of the Blastema

**Figure 3:**
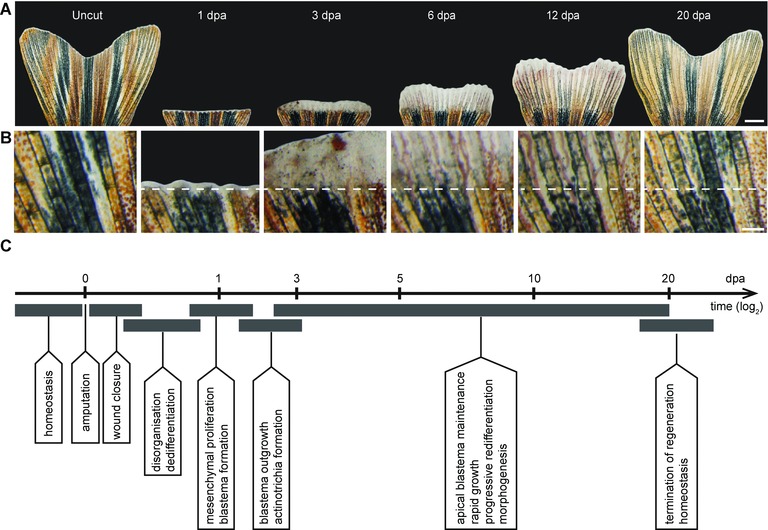
The regeneration process of the caudal fin in zebrafish. (A) Time‐lapse imaging of the same fin during the regeneration process at 27°C. Uncut, the original fin prior to amputation presents a bi‐lobed morphology. At 1 dpa, white tissue above the amputation consists of the wound epidermis and a few blastema cells. At 3 dpa, a white excrescence above the amputation plane contains the blastema, which, despite its uniform appearance, exhibits subdivisions at the cellular and molecular level. At 6 dpa, the outgrowth extends very rapidly; the white tissue is maintained at the fin margin, while the proximal outgrowth starts to display bone structures and pigmentation, which are the macroscopic markers of tissue redifferentiation. At 12 dpa, fin regeneration is at its advanced stage. At 20 dpa, the size of the fin nearly reaches its original size and pattern. The white margin of tissue remains at the tip for homeostatic growth/regeneration. (B) Higher magnifications of the fin surface at the position of amputation (white dashed line) at the respective time points are indicated in the upper panel (A). (C) The milestones of the fin regeneration process. Scale bars: (A) 1000 μm; (B) 200 μm.

The regenerating appendages of fish and amphibians are classified as examples of the epimorphic type of regeneration. This term refers to “the case of regeneration in which a proliferation of material precedes the development of the new part” (Morgan 1901). A highly proliferative tissue forms at the injury site and typically contains undifferentiated cells. This structure, called the blastema, can be observed with the naked eye and it was already reported in the first historical study on fin regeneration. Broussonet described the blastemal outgrowth in goldfish as “a kind of whitish excrescence (…) on the third day on the edge which had been cut” (Broussonet 1789). This unusual structure markedly elongates within a few days, and progressively replaces the missing part of the fin. Quoting Broussonet: “On the eighth day this excrescence was sensibly extended, and it soon became a membrane, which at first was only a line in breadth. This membrane (…) as it extended itself, it became thinner, and transparent” (Broussonet 1789). The historical description also fits the macroscopic appearance of the blastemal outgrowth in zebrafish (Fig. 3). In the case of the adult zebrafish caudal fin, the whitish stripe of tissue emerges beyond the amputation plane between the first and second day after amputation (Fig. 3A, B). Then, the regenerating structure enlarges and remains whitish until the third to fourth day. Starting from the fifth to sixth day, the white tissue persists only at the distal area of the outgrowth, while the proximal part of the new tissue progressively redifferentiates into the mature fin fold and acquires pigmentation. After approximately 3 weeks, the size and form of the fin is fully restored, even though a very thin whitish material is maintained at the fin margin throughout the entire life of the animal to account for ontogenetic growth and homeostatic tissue replacement throughout the lifespan.

Microscopic analyses of both fin and urodele limb regenerates revealed the cellular organization of the blastema as a cluster of undifferentiated proliferating cells covered by the wound epidermis (Brockes & Kumar 2002; Akimenko et al. 2003; Poss et al. 2003). One of the major challenges in fin/limb regeneration research was to determine the origin and potency of blastema cells. Although it was initially assumed that the blastema might be composed of a homogeneous and pluripotent cell population, this interpretation has been revised using detailed histological and immunohistochemical analyses (Steen 1970; Nechiporuk & Keating 2002; Santos‐Ruiz et al. 2002). Furthermore, recent genetic lineage tracing analysis revealed that cell fates in the blastema of fish and amphibians are restricted with respect to spatial and developmental identities under normal conditions (Knopf et al. 2011; Sousa et al. 2011; Tu & Johnson 2011; Singh et al. 2012; Stewart & Stankunas 2012). The fin blastema arises by migration and proliferation of fibroblasts followed by dedifferentiated osteoblasts (Fig. 2D). The relative position of both tissue types is preserved between the stump and the blastema. Specifically, the core of the blastema consists of a loose cluster of mesenchymal cells, while the undifferentiated osteoblasts maintain their original distribution underneath the wound epidermis, recapitulating the pattern of mature bones in the stump (Fig. 2E−G). Accordingly, the dedifferentiated migrating osteoblasts neither invade the interray tissue nor intermingle with the mesenchymal cells of the rays. In conclusion, the histological architecture of the blastema outgrowth displays a remarkable degree of spatial histological organization that reproduces the pattern of the original structures.

## Organizing Factors of the Blastema

The blastemal outgrowth represents a spatio‐temporally organized field of cells with the developmental plasticity for reconstruction of the missing parts. It remains a challenge to understand how such a developmentally potent structure can be formed de novo within a few days from the stump of the mature organ. A surgical cut obviously disrupts the status quo of the interconnected tissues, resulting in changes of the tensional and traction forces between the cells (Mammoto & Ingber 2010). Consequently, the elastic connective tissue is pulled away from the amputation plane, while epidermal cells are pushed beyond the wound margin towards the missing body part. Interestingly, the incision‐induced displacement of the epithelial cells occurs not only in the stump but also in the separated fin piece, which suggests a role of biophysical factors during the initial step of wound healing. Within the first day, the wound epidermis becomes thickened and the connective tissue within a distance of approximately 150 μm from the amputation plane undergoes disorganization (Fig. 2D) (Nechiporuk & Keating 2002). The fibroblasts of the activated mesenchyme round up, express tissue remodeling proteins, such as tenascin C, and start to proliferate (Jaźwińska et al. 2007). The early regeneration genes are induced to set up the two key structures of the regenerate, namely a specialized wound epithelium and the blastema, with proliferating cells of mesenchymal origin. The main unresolved question is how the tissue repair mechanism reactivates the regeneration program to generate both structures.

The epithelial−mesenchymal interactions are fundamental to the execution of developmental and regenerative programs (Yoshinari & Kawakami 2011; Blum & Begemann 2013; Gemberling et al. 2013). Accordingly, the wound epidermis functions not only as a physical barrier to protect the internal fin tissues, but also as an organizer of the underlying blastema. The latter function is attributed particularly to the basal layer of the wound epidermis that consists of a single row of aligned cells forming a niche‐like environment for the blastema. The wound epithelium provides architectural cues and secreted factors, such as Sonic hedghog (Shh), Wnt5b, Fgf24, to control blastema function (Laforest et al. 1998; Poss et al. 2000a,b; Quint et al. 2002; Lee et al. 2009). On the other hand, the formation of the specialized wound epithelium is dependent on the signals from the blastema, such as Fgf20a, Sdf1, Igf2b, and retinoic acid (RA) (Whitehead et al. 2005; Dufourcq & Vriz 2006; Bouzaffour et al. 2009; Chablais & Jazwinska 2010; Blum & Begemann 2012). The inhibition of any of these signaling pathways prevents both blastema formation and wound epithelium organization. The reciprocal communication between the wound epithelium and mesenchyme is also one of the prerequisites for blastema formation in the amphibian limb (Campbell & Crews 2008), indicating similar principles for appendage regeneration in vertebrates.

After the establishment of the interacting wound epithelium and blastema, cell proliferation takes place very rapidly and the increase of the outgrowth size has to be immediately accompanied by pattern formation. Accordingly, the wound epithelium and the blastema acquire a proximo‐distal specification starting at 3 days post‐amputation (dpa), which can be well distinguished on the longitudinal fin sections (Fig. 2E). The apical part of the outgrowth is formed by a columnar basal epithelium and the distal‐most blastema, which comprises mesenchymal cells with a slow proliferative activity (Nechiporuk & Keating 2002). Importantly, the distal‐most blastema is restricted exclusively to the very tip of the blastema. In situ hybridization analyses demonstrated that several genes, such as *aldh1a2*, *wnt5a*, *fgf3*, demarcate a broader extent of the distal blastema, including rapidly proliferating cells (Stoick‐Cooper et al. 2007; Mathew et al. 2009; Stewart et al. 2014; Wehner et al. 2014). The proximal compartment of the blastema comprises a central cluster of rapidly proliferating mesenchymal cells and the lateral compact layers of dedifferentiated osteoblasts that are located underneath the cuboidal basal wound epithelium. The cuboidal wound epithelium expresses a signaling protein, Shh, that could be involved in guidance of the underlying osteoblasts through the regeneration process (Laforest et al. 1998; Smith et al. 2006; Zhang et al. 2012). However, the interdependence between the wound epidermis and osteoblast differentiation remains speculative, and this interesting topic requires further study. Thus, the wound epithelium and the blastema display a compartmentalization already at the early outgrowth phase. It has been proposed that the apical part of the blastema acts as the upstream organizer of the regenerate through the Wnt signaling pathway, which regulates epidermal patterning, blastemal cell proliferation, and osteoblast maturation indirectly via secondary signals, such as Fgf and bone morphogenetic protein (BMP) (Wehner et al. 2014). On the other hand the proximal compartment of the blastema has a regenerative task to maintain high cell proliferation and their progressive redifferentiation. Recently, two studies have reported that a balance between the two processes is regulated by the Notch signaling pathway (Grotek et al. 2013; Münch et al. 2013). Inhibition of Notch signaling reduces blastema cell proliferation and results in a complete block of fin regeneration. In contrast, overactivation of Notch signaling leads to a proximal expansion of the proliferative blastema zone and the inhibition of osteoblast differentiation. Thus, the Notch signaling pathway seems to be activated in the blastema during regenerative outgrowth to maintain blastemal cells in a proliferative and undifferentiated state and to inhibit terminal differentiation (Grotek et al. 2013; Münch et al. 2013). It still remains a challenge to understand how various signaling pathways are integrated to achieve the functional subdivision of the blastema and wound epithelium.

During the outgrowth phase, the blastema becomes vascularized and innervated. Blocking angiogenesis through inhibition of vascular endothelial growth factor receptor does not impair the initial wound epidermis and blastema formation (Bayliss et al. 2006). In the absence of blood supply within the regenerate, the elongation of the outgrowth is terminated at approximately 3 dpa. Although the role of innervation during blastema formation has been extensively investigated in the amphibian limb, little is known about this topic in the context of the fin. The importance of nerves during pectoral fin regeneration has been reported in a study in *Fundulus* fish and in a recent study in zebrafish (Geraudie & Singer 1978; Simões et al. 2014). However, evidence for the requirement of nerves during blastema formation in the zebrafish caudal fin is still missing.

## Regeneration of the Fin Skeleton

In the absence of muscles, the skeleton represents the main structure that supports the function of the fin as a locomotory appendage. The rays display patterning along the proximo‐distal axis of the fin. The unique feature of the distal segments, which makes them distinct from the proximal ones, is the presence of the actinotrichia, non‐mineralized spicules organized in brush‐like bundles (Fig. 1C) (Durán et al. 2011). Actinotrichia‐specific genes, such as *actinodin‐1*, are transcriptionally induced during the early blastema outgrowth phase, suggesting that the early dedifferentiated mesenchymal cells acquire the distal‐most identity (Zhang et al. 2010; A. Jaźwińska, unpubl. data). Thus, blastema formation is associated with the reversion from the proximal to distal identity, which represents an opposing transformation compared to the addition of ray segments during ontogenetic growth. At this point, it is worth emphasizing the remarkable plasticity of the adult fin tissue to transiently activate and suppress the distal‐specific genes depending on the real‐time position of the fin margin. Based on actinotrichia morphogenesis in the blastema, the early regenerative outgrowth phase involves by default the re‐establishment of the distal structures of the ray. The molecular mechanisms that combine the spatial recognition of the fin margin with the transient determination of the distal structures have not yet been investigated.

During fin regeneration, actinotrichia are formed within 48 h post‐amputation (hpa). At 3 dpa, the thickest bundles of actinotrichia accumulate between the wound epidermis and the blastema, while the fine actinotrichial fibers build a mash between the mesenchymal cells (Pfefferli et al. 2014). Thus, the actinotrichia arise as the first skeletal support for the proliferating mesenchymal cells within the membrane‐like outgrowth, prior to the bone matrix. Although the deposition of lepidotrichial tissue requires more time than for actinotrichia, the redifferentiation of the bone can be observed at the cellular level using transgenic reporter lines, such as *runx2* for pre‐osteoblasts, *osterix* (*sp7*) for intermediately differentiated (committed) osteoblasts, and finally *osteocalcin* for fully differentiated bone‐forming cells (Knopf et al. 2011). The application of the set of transgenic fish lines provides a tool to examine the dynamics of the differentiation process during regeneration. Recently, it has been proposed that generation and maintenance of proliferative *runx2*‐positive pre‐osteoblasts is controlled by Wnt/β‐catenin signaling at the distal tip of the regenerate (Stewart et al. 2014). The redifferentiation of osteoblast progenitor cells in the proximal compartment requires downregulation of Wnt activity by BMP signaling via induction of Wnt antagonists. Thus, the interplay between the two signaling pathways coordinates dedifferentiation and redifferentiation of osteoblasts (Stewart et al. 2014). However, the role of Wnt signaling in bone regeneration seems to be more complex, as another study reported an indirect role of Wnt signaling in the regulation of osteoblast differentiation through actinotrichia‐forming cells (Wehner et al. 2014). Thus, the signaling pathways promoting actinotrichia formation remain to be elucidated.

Cell lineage tracing experiments combined with transgenic technologies in zebrafish showed that the regenerated tissues derive from pre‐existing cells that retain their developmental identity during their transition in the blastema (Knopf et al. 2011; Sousa et al. 2011; Tu & Johnson 2011; Singh et al. 2012; Stewart & Stankunas 2012). However, this lineage commitment displays remarkable plasticity under certain restrictive conditions. The genetic ablation of all osteoblasts using a nitroreductase system did not prevent bone regeneration (Singh et al. 2012). This unexpected finding reveals an impressive plasticity of the fin to activate alternative mechanisms in order to generate de novo osteoblasts. Mosaic transgene expression analysis provides no evidence for a contribution by circulating stem cells to the fin regenerate (Tu & Johnson 2011). Thus the new osteoblasts could derive either from putative osteoblast stem cells or through transdifferentiation of mesenchymal blastema cells into bone‐forming cells. The latter explanation would involve the reactivation of developmental programs that promote osteoblast formation from the mesenchymal condensations (Grandel & Schulte‐Merker 1998). The recapitulation of developmental processes might be dependent on the activity of the Shh and BMP signaling pathways, which have been implicated in bone regeneration (Quint et al. 2002). Further studies are needed to understand the mechanisms controlling bone regeneration under normal and specific circumstances.

## Epigenetic Regulators of Fin Regeneration

Animals with extensive regenerative capacities are characterized by their ability to rapidly reactivate a large array of genes initially expressed during embryonic development. Their ability to maintain access to the developmental programs in the adult organism may correlate with the plasticity of their epigenome (Katsuyama & Paro 2011). The epigenetic constraints could also explain why some species have lost the capacity to regenerate during evolution. This topic has been recently investigated in the context of zebrafish caudal fin regeneration. Based on the assessment of 5‐methylcytosine and 5‐hydromethylcytosine, it has been proposed that the early phase of fin regeneration is characterized by a transient DNA demethylation and expression of DNA demethylation‐ and repair‐related genes (Hirose et al. 2013). The study of Stewart et al. (2012) demonstrated that histone modifications at specific loci might be an important regulatory mechanism for the reactivation of a regeneration gene expression program and for the initiation of regeneration. This study suggested that common developmental and regeneration genes are maintained in a dormant/silent flexible chromatin state in the adult caudal fin in zebrafish. The demethylation of the repressive mark H3K27me3 contributes to the regenerative response of the caudal fin after amputation. Our laboratory identified that specific epigenetic factors are required for the redifferentiation phase of regeneration (Pfefferli et al. 2014). Several components of the nucleosome remodeling and deacetylase (NuRD) complex, such as *chd4a*, *hdac1*, *rbb4*, and *mta2*, are transcriptionally upregulated in the proliferative compartment of the blastema, where cells also make a transition to a differentiated state. Chemical inhibition of the histone deacetylase 1 (Hdac1) does not interfere with initial blastema formation and osteoblast dedifferentiation, but leads to a blockage of redifferentiation of skeletal precursors and actinotrichia formation. This study suggests that, in the absence of a functional NuRD complex, blastema cells might be arrested in an undifferentiated or partially differentiated state, probably because of a failure in the activation of the morphogenesis program.

## Growth and Morphogenesis of the Fin Regenerate

The accuracy of appendage restoration in amphibians and fish immediately raises a question about the nature of factors that control the growth and morphogenesis of the missing parts in a precise three‐dimensional pattern. In this context, the discovery of the patterning defects induced by exogenously administered retinoids, including RA, gave important clues about the proximo‐distal axis specification in the regenerating limb (Brockes & Kumar 2002; Maden & Hind 2003). The classical studies in amphibians demonstrated that RA treatment triggers a duplication of the proximal bones prior to replacement of the amputated distal parts, resulting in abnormally long limbs (Maden 1982; Thoms & Stocum 1984). The interpretation of this effect was that an exposure to a higher concentration of RA is sufficient to re‐specify positional information along the amphibian limb axis in a proximal direction (Maden & Hind 2003). Disappointingly, such results could not be reproduced for the zebrafish caudal fin. RA treatment for several days followed by transfer to normal conditions does not induce formation of extra‐long fin regenerates, but causes a teratogenic effect probably due to massive cell death, predominantly in the epidermis (White et al. 1994; Ferretti & Géraudie 1995; Géraudie et al. 1995). The severity of the defects is dependent on the RA concentration, duration and time‐window of the treatment. In all conditions, RA causes narrowing of the medial−lateral axis of the regenerate by decreasing the amount of soft tissue between adjacent rays. This may lead in some cases to ray fusion, which occurs, importantly, without affecting the length of the regenerated rays. The differential outcomes of RA treatment in the amphibian limb and the caudal fin can be explained by the existence of divergent developmental strategies for the elongation of the extremities during growth, as compared above.

The dream of manipulating the robust regenerative patterning and of finding a substance that can induce extra‐long fin regenerates was only recently realized. Kujawski and colleagues (2014) identified a chemical, called tacrolimus (FK506), which can stimulate overgrowth of the fin not only during regeneration but also during homeostasis. This drug belongs to the category of immunosuppressants that act through inhibition of the protein phosphatase calcineurin. The treatment with FK506 during regeneration results in increased blastemal cell proliferation and ray elongation, indicating that calcineurin activity is required to slow down and to terminate regeneration (Kujawski et al. 2014). Bones of FK506‐treated regenerates display a distal shift of the bifurcation points, suggesting a change in positional information. Importantly, the increased fin size is not accompanied by extension of the main body. The authors suggest that calcineurin acts as a negative regulator of tissue growth along the proximo‐distal axis of the fin. It will be interesting to investigate whether this protein phosphatase has other morphogenetic functions, such as patterning of the ray segmentation, which is a stereotypic feature of the skeletal organization in the fin fold.

The factors controlling fin regrowth and morphogenesis can also be studied using a genetic approach in zebrafish. Several mutants have been identified that carry abnormally developed fins, some of which also display regeneration defects (van Eeden et al. 1996). One of these mutants, called *another long fin* (*alf^*dty86*^*), attracted much attention in research due to its extraordinarily elongated fins (Sims et al. 2009). The severity of the *alf* mutant phenotype is associated with skeletal defects of the fin, such as irregular and longer segments of the rays and misaligned joints. A lower frequency of elastic ligaments along the ray length was predicted to decrease the flexibility of the fin during swimming, leading to incidences of bone fractures and bone dislocation (Sims et al. 2009). The *alf* mutant locus has recently been identified as a gain‐of‐function mutation in *kcnk5b*, a gene encoding a two‐pore domain potassium channel, which probably causes hyperpolarization of the cell (Perathoner et al. 2014). The authors suggest that a coordinated ion flux may provide some cues for coordination of growth. A concept of molecular bioelectricity has already been implicated in diverse examples of regeneration, development, and oncogenesis (Levin 2014). The remaining question is how the bioelectrical signals regulate downstream cellular responses to determine positional information and to induce morphogenetic decisions such as segmental border formation.

The opposite phenotype to the *alf* elongated fins is represented by another genetic mutation called *shortfin* (*sof ^*b123*^*) that causes shortened ray segments and shorter fins compared to wild type (Iovine et al. 2005). *sof* mutants exhibit a decreased expression of *connexin 43* (*cx43*), a component of gap junctions, as opposed to *alf* mutants with enhanced levels of Cx43 (Hoptak‐Solga et al. 2007; Sims et al. 2009). The gap junctions serve as stimuli‐regulated intercellular channels for sharing small molecules, such as inorganic ions and metabolites, during development and homeostasis (Goodenough et al. 1996; Kumar & Gilula 1996; Ton & Iovine 2013). Thus, both opposing fin‐size phenotypes of *alf* and *sof* mutants are associated with aberrant membrane channels involved in ion flux. A loss of Cx43 activity leads to short segments, while a gain of Kcnk5b and Cx43 activities results in longer segments. It becomes evident that the ion flux in a cluster of proliferating cells is essential to orchestrate morphogenetic decisions during development and regeneration.

Transcriptome analyses have been performed to identify the downstream effectors of the *sof* and *alf* mutations (Ton & Iovine 2012). One of the selected candidate genes is *semaphorin 3d* (*sema3d*), which belongs to the family of secreted ligands that interact with cell surface receptors to regulate adhesion, migration, and proliferation (Yazdani & Terman 2006). *sema3d* is expressed in a subdomain of the basal layer composed of cuboidal‐shaped cells in the wound epidermis, as well as in the adjacent skeletal precursors in the blastema (Ton & Iovine 2012). Thus, *sema3d* expression does not overlap with the Cx43 domain in the mesenchyme of the blastema (Hoptak‐Solga et al. 2008). Two models have been proposed to explain how intercellular communication through the Cx43‐dependent gap junction in mesenchymal cells influences *sema3d* gene expression in the adjacent osteoblasts (Ton & Iovine 2013). The first hypothesis relies on the secondary unidentified signal between the mesenchyme and osteoblasts, while the second option postulates direct communication between both tissues via heterotypic gap junctions that would be composed of different connexin proteins. Further studies of intercellular communication between different cell populations of the regenerate will bring novel insights about the coordination of growth and morphogenesis.

## Conclusions and Perspectives

The historical study identified that a formation of “whitish excrescence” precedes morphogenesis of the fin regenerate. In the course of the last decades, it has been recognized that this structure contains the blastema, a heterogeneous group of dedifferentiated/proliferative cells that are capable of regrowing and patterning a complex organ. Application of genetic and chemical tools demonstrated that several canonical signaling pathways are required for blastema formation and function. Recent studies highlighted the importance of ion concentration, gap‐junction‐mediated intercellular communication and protein phosphatase activity, to regulate a specific gene expression program and cellular behavior. Little is known about the mechanisms that render the differentiated cells responsive to the regeneration signals. What makes the adult zebrafish cells so plastic to reactivate the fin‐specific developmental program upon signaling? What mechanisms allow the adult cells to rapidly switch between dedifferentiation and redifferentiation, proliferation and morphogenesis, proximal and distal identity during regeneration? There is certainly a correlation between the epigenetic chromatin status of cells and the responsiveness to the developmental cues. One of the big challenges remains to identify the factors that regulate this cellular plasticity in vertebrate organisms capable of regeneration.
